# Differential Influence of Age on the Relationship between Genetic Mismatch and A(H1N1)pdm09 Vaccine Effectiveness

**DOI:** 10.3390/v13040619

**Published:** 2021-04-04

**Authors:** Lirong Cao, Shi Zhao, Jingzhi Lou, Hong Zheng, Renee W. Y. Chan, Marc K. C. Chong, Zigui Chen, Paul K. S. Chan, Benny C. Y. Zee, Maggie H. Wang

**Affiliations:** 1 JC School of Public Health and Primary Care, Chinese University of Hong Kong, Shatin, N.T., Hong Kong, China; caolr@link.cuhk.edu.hk (L.C.); zhaoshi.cmsa@gmail.com (S.Z.); jzl@bethbio.com (J.L.); hongzheng@cuhk.edu.hk (H.Z.); marc@cuhk.edu.hk (M.K.C.C.); 2 CUHK Shenzhen Research Institute, Shenzhen 518000, China; 3 Hong Kong Hub of Paediatric Excellence, Chinese University of Hong Kong, Shatin, N.T., Hong Kong, China; reneewy@cuhk.edu.hk; 4 Department of Pediatrics, Chinese University of Hong Kong, Shatin, N.T., Hong Kong, China; 5 CUHK-UMCU Joint Research Laboratory of Respiratory Virus & Immunobiology, Chinese University of Hong Kong, Shatin, N.T., Hong Kong, China; 6 Li Ka Shing Institute of Health Sciences, Faculty of Medicine, Chinese University of Hong Kong, Shatin, N.T., Hong Kong, China; 7 Department of Microbiology, Stanley Ho Centre for Emerging Infectious Diseases, Li Ka Shing Institute of Health Sciences, Chinese University of Hong Kong, Shatin, N.T., Hong Kong, China; zigui.chen@cuhk.edu.hk (Z.C.); paulkschan@cuhk.edu.hk (P.K.S.C.)

**Keywords:** A(H1N1)pdm09, influenza vaccine effectiveness, age-related effects, genetic mismatch

## Abstract

Assessment of influenza vaccine effectiveness (VE) and identification of relevant influencing factors are the current priorities for optimizing vaccines to reduce the impacts of influenza. To date, how the difference between epidemic strains and vaccine strains at genetic scale affects age-specific vaccine performance remains ambiguous. This study investigated the association between genetic mismatch on hemagglutinin and neuraminidase genes and A(H1N1)pdm09 VE in different age groups with a novel computational approach. We found significant linear relationships between VE and genetic mismatch in children, young adults, and middle-aged adults. In the children’s group, each 3-key amino acid mutation was associated with an average of 10% decrease in vaccine effectiveness in a given epidemic season, and genetic mismatch exerted no influence on VE for the elderly group. We demonstrated that present vaccines were most effective for children, while protection for the elderly was reduced and indifferent to vaccine component updates. Modeling such relationships is practical to inform timely evaluation of VE in different groups of populations during mass vaccination and may inform age-specific vaccination regimens.

## 1. Introduction

Since the emergence of the pandemic H1N1 influenza in early 2009, the swine-origin A(H1N1)pdm09 virus has replaced the seasonal H1N1 subtype and become one of the four major circulating viruses underlying seasonal influenza epidemics [1]. The new H1N1 pandemic virus is antigenically distinct from the former seasonal H1N1 virus [2]. In response, the new subtype is introduced as one of the components in the trivalent or quadrivalent seasonal influenza vaccines [3]. As influenza viruses are evolving rapidly, the World Health Organization (WHO) annually reviews and recommends influenza vaccine compositions for vaccine updates.

Typically, vaccine effectiveness (VE) estimation is conducted by retrospective investigation in the middle and late stage during epidemics or at the end of the flu season [4,5]. The current VE exhibits high season-to-season variations, mostly due to the genetic mismatch of the circulating strains and the vaccine virus [6]. Apart from survey-based VE studies, the hemagglutination inhibition (HI) test is a standard method to evaluate the antigenic distance [7]. The HI test detects the prevention of binding of the epitopes in the hemagglutinin protein to the sialic acid receptors on red blood cells [8]. Studies based on antigenic distance showed that past vaccinations might have a negative or positive interference on current vaccine efficacy depending on the antigenic relatedness among the previous vaccine virus, current vaccine virus and the circulating strains [9,10,11]. Sequence-based methods were also proposed to estimate the VE using various genetic mismatch summary statistics [12,13]. These sequence-based studies showed a clear trend that a weaker VE is associated with a larger genetic mismatch of the circulating strains and vaccine viruses. However, the existing genetic-VE relationship is developed for the all-age group. Although it is clear that the seniority is associated with a reduced vaccine-induced immune response [14,15,16,17], it is unclear whether genetic mismatch will generate a similar VE response in age generations. Therefore, in this study, based on the previous statistical framework [13], we quantified the effect of genetic mismatch on observed VE in different age groups.

## 2. Materials and Methods

### 2.1. Genetic Data

Protein sequences of human influenza A(H1N1)pdm09 virus were retrieved from the global initiative on sharing all influenza data (GISAID) [18], with sampling dates ranging from 1 January 2009 to 31 December 2019. The sequences with age information of the host were collected. We stratified the samples into five groups according to the commonly adopted scheme in VE studies, namely: (1) children: age ≤ 8 years; (2) adolescents: 8 years < age ≤ 17 years; (3) young adults: 17 years < age ≤ 49 years; (4) middle-aged adults: 49 years < age ≤ 64 years; and (5) the elderly: age > 64 years [5]. Duplicated strains were removed. For the model building samples, in total 5232 hemagglutinin (HA) and neuraminidase (NA) strains were retained with sample origins of the United States and Canada. The independent validation set included a total number of 3470 strains from the United Kingdom, Germany, Spain, Italy, France, Greece, Sweden, Mexico, Japan, and Hong Kong. Multiple sequence alignment was performed with Clustal X [19]. The detailed sample size of genetic data is shown in Supplementary Materials Table S1.1. All sequence data used in the analysis are acknowledged in the Supplementary Acknowledgment Table.

### 2.2. Vaccine Effectiveness Data

The VE data were collected from published studies in the target countries. The inclusion criteria of the studies were: (1) VE evaluation (or corresponding odds ratio) was reported for A(H1N1)pdm09; (2) original research of VE was conducted with a test-negative design by observational studies; (3) RT-PCR method was used to confirm the positive status of infection; (4) patients were recruited by predefined illness criteria; and (5) age-stratified VE outcomes were reported. The exclusion criteria included: (1) the study was restricted to a population with special conditions such as asthma patients or pregnant women; (2) interim reports and, if relevant, final reports were available. The eligible studies between 2009 and 2020 are listed in Supplementary Materials Table S1.2.

### 2.3. Statistical Methods

In previous research, we proposed the effective mutation distance (EMD) as a summary statistic to evaluate genetic mismatch between vaccine strains and circulating strains [13]. The EMD was defined by the Hamming distance on the effective mutations (EMs) underlying seasonal influenza epidemics [20,21,22], which could be interpreted as the amino acid substitutions that contributed to the viral escape from herd immunity. Thus, the EMs served as a rational basis to evaluate the genetic distance associated with vaccine response. The list of EM sites for the A(H1N1)pdm09 can be found in Supplementary Materials Table S1.3. We calculated EMDs for HA and NA genes separately in each epidemic season [13]. The generalized linear regression model was fitted to evaluate the relationship between the EMD and the VEs for each age group. Repeated-measures analysis of variance (ANOVA) and the Friedman test were respectively used to assess the difference in observed VE and genetic measure across age groups. Paired *t*-test and Wilcoxon signed-rank test were used to perform pairwise comparisons. Statistical significance was declared if *p*-value < 0.05. All analyses were conducted using R statistical software (version 4.0.3) [23].

## 3. Results

### VE, EMD and Genetic Effect Size across Age Groups

**Figure 1:**
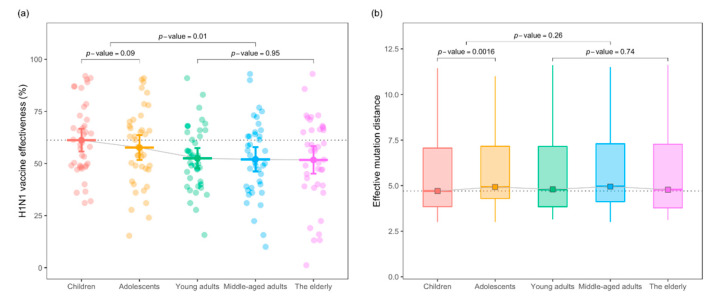
Comparison of H1N1 vaccine effectiveness (VE) and effective mutation distance (EMD) among age groups. Panel (**a**): distribution of the observed VE in different age groups. VE in the younger groups was higher than adults (paired *t*-test *p*-value = 0.01). Panel (**b**): EMD distribution in different generations. No statistical difference in EMD between the younger groups and the adult groups is present (Wilcoxon test *p*-value = 0.26). In panels (**a**) and (**b**), dashed lines represent the mean observed VE and the median EMD of the children’s group.

**Figure 2:**
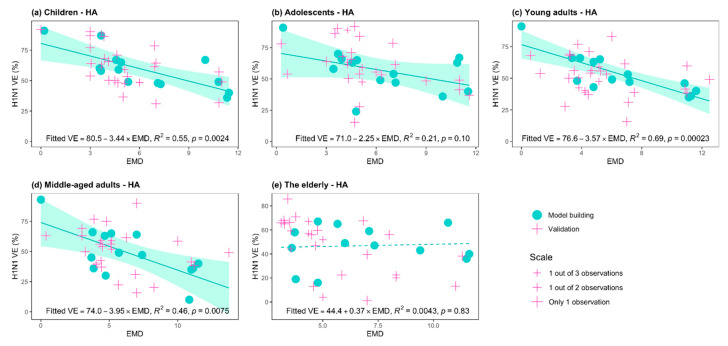
The relationship between vaccine effectiveness (VE) of A(H1N1)pdm09 and effective mutation distance (EMD) on hemagglutinin (HA) gene, in different age groups: (**a**) children; (**b**) adolescents; (**c**) young adults; (**d**) middle-aged adults; (**e**) the elderly. Green dots: model building samples from the United States and Canada; pink cross: independent validation samples from the United Kingdom, Germany, Spain, Italy, France, Greece, Sweden, Mexico, Japan, and Hong Kong. In the first four age groups, clear linear relationships were observed between VE and the EMD; the validation samples largely followed the relationship estimated by the North American samples (**a**–**d**). In the elderly population, no statistically significant association is present between VE and the EMD.

First, we compared the observed VE and genetic mismatch of the isolated viruses to the vaccine strain in all age groups. As expected, the observed VEs of the young adults, middle-aged and elderly groups were significantly lower compared with the children and adolescent groups (*p*-value = 0.01 for two-sided paired *t*-test, see Figure 1a). Secondly, the genetic measure in terms of the EMD of the HA gene showed no statistically significant difference between the adults and the younger age groups (*p*-value = 0.26 for Wilcoxon test, Figure 1b). Next, we conducted linear regression analysis within each age stratum, and the effect size of the HA-EMD inferred per key substitution’s associated reduction in VE. Figure 2 plots the age-stratified linear relationship between the EMD and VE. In the children, young adults and middle-aged groups, the estimated per substitution effect on the reduction of VE was 3.44%, 3.57%, and 3.95% in absolute terms, respectively (*p*-values < 0.01, Figure 2a,c,d). That is to say, every three key amino acid mutations will induce a decrease in vaccine effectiveness by an average of ~10% in a given epidemic season. For adolescents, HA mismatch was weakly associated with VE, and per mismatch yielded an average of a 2.25% decrease in VE (*p*-value = 0.10, Figure 2b). We found that genetic mismatch exerted no influence on VE for the elderly group (*p*-value = 0.83, Figure 2e).

In each age group, independent validation data points were overlaid on the estimated relationship between HA-EMD and VE (Figure 2). In the children, young adults and middle-aged groups in which the genetic effect was significant, validation data showed close clustering around the predicted line, with an average mean absolute error (MAE) of 13.4%. The intercept of the fitted regression implied the highest VE achievable for existing influenza H1N1 vaccines when there was no genetic mismatch on key loci (EMD = 0), the range of which was covered by the observed data. Figure 2 showed that the children’s group would result in a maximum VE of 80.5% [95% CI: 67.9–93.1%] if a perfect match of vaccine virus is present, while this optimal value was slightly lower, 76.6% [95% CI: 66.7–86.5%], for the young adults and 74.0% [95% CI: 56.4–91.6%] for the middle-aged adult group, respectively. The highest VE was 71.0% [95%CI: 53.1–88.9%] for the adolescent group. In the elderly population, no linear relationship was observed between genetic mismatch and VE, and the grand mean of VE was 44.4% [95% CI: 19.8–69.0%], regardless of the matching level between the vaccines and circulating strains.

We also conducted analysis using the EMD of the NA gene and VE (Supplementary Materials Figure S2.1). NA-EMD showed a generally consistent pattern of effect size distribution, in which the children, young adults, and the middle-aged groups had the largest genetic effect size on VE, while the VE of the elderly group was not related to vaccine matching levels. In general, the magnitudes of NA genetic effect were lower compared with HA-EMD.

## 4. Discussion

This study explored the role of genetic mismatch on vaccine performance in different age groups for the influenza A(H1N1)pdm09 virus. We identified a significant negative association between the genetic mismatch measure and observed VE among the children, young adults, and middle-aged groups. We reported a weak association in the adolescent group, and the relationship does not appear statistically evident in the elderly group. For an ideal genetically matched vaccine, the 0–8 years old group would exhibit the highest vaccine protection effect, while the age > 64 years group would not show differential response to the alterations of vaccine components.

Our findings characterized impaired immunity in the elderly population by the relationship between genetic mismatch and vaccine response. Previous studies reported relatively lower vaccine performance in the senior group [15,16]. One possible explanation is immunosenescence: a decline in the immune function affects the susceptibility to influenza infection and responds to administered vaccines during aging [24]. The response to influenza vaccines could also be shaped by childhood immune imprinting of the H1N1 viruses or the original antigenic sin, such that complicated immune histories of the elderly limit the protection of vaccination [25,26,27]. Special vaccination schemes, e.g., the use of vaccine adjuvant, increased dosage, or repeated vaccination, shall be implemented to enhance vaccine protection in the senior population [28,29].

The same explanation also applies to the children’s group, in which the highest antibody response to the virus-matched vaccine is identified. A large proportion of this population has experienced the primary vaccination during the period and thus generated the strongest antigen-specific immune responses [30], while broader recall responses are mounted in adults [31]. Consequently, adolescent individuals, after the development of primary immunity against H1N1 in childhood, might have suffered the original antigenic sin as similar H1N1 viruses presented as secondary infections. Research also reported that the VE of adolescents was much lower than other age groups during the same flu season [5,32], which may be due to the inability of pre-existing cross-reactive antibodies and vaccine-elicited antibodies to provide adequate protection [33]. Young adults and middle-aged individuals, age range 18–64 years, might have undergone less frequent vaccination compared with children [17] and might be infected by relatively distant genetic variants compared with prior antigens, and thus vaccine-induced immunity showed stronger responses to the matching of the vaccine virus. Further investigation is needed to explore whether variations in pre-exposure histories, vaccine doses, or vaccine types caused this difference.

The main limitation of this study is that only genetic factors were considered in the analysis, and in-host immunological measurements were not included as a constraint by limited resources.

## 5. Conclusions

We demonstrated that influenza vaccine effectiveness was evidently enhanced in non-elderly groups when genetic matching was improved, while vaccine protection for the elderly was generally lower and indifferent to vaccine component updates. Through evaluating the genetics and vaccine response relationship, this study indicates the importance of optimizing vaccine components to match circulating viruses, and a special vaccination scheme is needed to increase the protection of the elderly population.

## Data Availability

Publicly available datasets were analyzed in this study. This data can be found in the Supplementary Acknowledgment Table.

## Supplementary Materials

The following are available online at https://www.mdpi.com/article/10.3390/v13040619/s1. Table S1.1: Genetic data sample size-number of strains. Table S1.2: Influenza A(H1N1)pdm09 vaccine effectiveness in Northern Hemisphere, 2009-2019. Table S1.3: List of Effective Mutation (EM) sites on HA and NA genes of A(H1N1)pdm09. Figure S2.1: The relationship between H1N1 VE and EMD on neuraminidase (NA), in different age groups.
