# Supplementary material for: Differential Influence of Age on the Relationship between Genetic Mismatch and A(H1N1)pdm09 Vaccine Effectiveness
Source: Viruses. 2021 Apr 4;13(4):619. doi: 10.3390/v13040619 (PMC8065480; doi:10.3390/v13040619)
Supplement: Supplementary file 1 [file viruses-13-00619-s001.zip › viruses-1155943-supplementary/Supplementary Materials.docx]

**Supplementary Materials**

Table of Contents

[S1 Supplementary tables 2](#_Toc68379550)

[Table S1.1. Genetic data sample size - number of strains 2](#_Toc68379551)

[Table S1.2. Influenza A(H1N1)pdm09 vaccine effectiveness in Northern Hemisphere, 2009 - 2019 3](#_Toc68379552)

[Table S1.3. List of Effective Mutation (EM) sites on HA and NA genes of A(H1N1)pdm09 5](#_Toc68379553)

[S2 Supplementary figure 6](#_Toc68379554)

[Figure S2.1. The relationship between H1N1 VE and EMD on neuraminidase (NA), in different age groups. 6](#_Toc68379555)

[References 7](#_Toc68379556)

**S1 Supplementary tables**

## Table S1.1. Genetic data sample size - number of strains

| Age groups | Sample size for model building set | |  | Sample size for validation set | | Total |
| --- | --- | --- | --- | --- | --- | --- |
|  | No. of HA^*^ strains | No. of NA^**^ strains |  | No. of HA strains | No. of NA strains |  |
| Children | 545 | 542 |  | 589 | 337 | 2013 |
| Adolescents | 373 | 373 |  | 209 | 146 | 1101 |
| Yong adults | 756 | 754 |  | 784 | 404 | 2698 |
| Middle-aged adults | 527 | 523 |  | 258 | 157 | 1465 |
| The elderly | 420 | 419 |  | 416 | 170 | 1425 |

*HA: hemagglutinin; **NA: neuraminidase

## Table S1.2. Influenza A(H1N1)pdm09 vaccine effectiveness in Northern Hemisphere, 2009 - 2019

| No. | Countries or regions | Flu season | Source |
| --- | --- | --- | --- |
| 1 | United States | 2010-11 | US CDC [1] |
| 2 | United States | 2011-12 | US CDC [2] |
| 3 | United States | 2012-13 | US CDC [3] |
| 4 | United States | 2013-14 | US CDC [4] |
| 5 | United States | 2014-15 | US CDC [5] |
| 6 | United States | 2015-16 | US CDC [6] |
| 7 | United States | 2016-17 | US CDC [7] |
| 8 | United States | 2017-18 | US CDC [8] |
| 9 | United States | 2018-19 | US CDC [9] |
| 10 | Canada | 2009-10 | BC CDC [10] |
| 11 | Canada | 2015-16 | BC CDC [11] |
| 12 | Canada | 2016-17 | BC CDC [12] |
| 13 | Canada | 2017-18 | BC CDC [12] |
| 14 | Canada | 2018-19 | BC CDC [13] |
| 15 | United Kingdom | 2010-11 | Pebody RG 2013 [14] |
| 16 | United Kingdom | 2012-13 | Andrews N 2014 [15] |
| 17 | United Kingdom | 2015-16 | Pebody RG 2016 [16] |
| 18 | United Kingdom | 2015-16 | Pebody RG 2017 [17] |
| 19 | United Kingdom | 2017-18 | GOV UK [18] |
| 20 | United Kingdom | 2018-19 | Kissling E 2019 [19] |
| 21 | United Kingdom | 2018-19 | Pebody RG 2020 [20] |
| 22 | United Kingdom | 2018-19 | Pebody RG 2020 [21] |
| 23 | Germany | 2010-11 | EUR CDC [22] |
| 24 | Germany | 2012-13 | EUR CDC [23] |
| 25 | Germany | 2013-14 | EUR CDC [24] |
| 26 | Germany | 2015-16 | EUR CDC [25] |
| 27 | Germany | 2017-18 | EUR CDC [26] |
| 28 | Germany | 2018-19 | EUR CDC [19] |
| 29 | Spain | 2010-11 | Kissling E 2016 [27] |
| 30 | Spain | 2012-13 | Kissling E 2016 [27] |
| 31 | Spain | 2012-13 | Rondy, M [28] |
| 32 | Spain | 2013-14 | Kissling E 2016 [27] |
| 33 | Spain | 2013-14 | Castilla, J [29] |
| 34 | Spain | 2014-15 | Kissling E 2016 [27] |
| 35 | Spain | 2015-16 | Puig-Barberà J [30] |
| 36 | Spain | 2015-16 | Rondy M [31] |
| 37 | Spain | 2017-18 | Kissling E 2019 [32] |
| 38 | Spain | 2018-19 | Castilla J 2020 [33] |
| 39 | Italy | 2014-15 | Rizzo C [34] |
| 40 | Italy | 2017-18 | Bella A [35] |
| 41 | Italy | 2018-19 | Bellino S [36] |
| 42 | France | 2013-14 | Rondy M [37] |
| 43 | France | 2016-17 | Souty C [38] |
| 44 | Greece | 2013-14 | Lytras T [39] |
| 45 | Sweden | 2010-11 | Widgren K [40] |
| 46 | Mexico | 2016-17 | Castillejos M [41] |
| 47 | Japan | 2018-19 | Ando S [42] |
| 48 | Hong Kong | 2017-18 | Chan YD 2019 [43] |
| 49 | Hong Kong | 2018-19 | Chiu S [44] |

## Table S1.3. List of Effective Mutation (EM) sites on HA and NA genes of A(H1N1)pdm09

| Gene | # of EM sites | EM sites list |
| --- | --- | --- |
| Hemagglutinin (HA) | 17 | 13, 91, 101, 114, 160, 179, 180, 181, 200, 202, 214, 233, 273, 300, 312, 391, 468. |
| Neuraminidase (NA) | 16 | 13, 34, 40, 44, 77, 81, 106, 188, 200, 264, 270, 314, 321, 386, 432, 449. |

Legend: All Effective Mutation (EM) sites involved in this study were identified by the previous research [45-48]. The EM sites are identified based on two criteria: the prevalence of amino acid substitution achieving dominance threshold in a time interval; and the duration of the occurrence of such ‘dominant’ substitutions. If the total prevalence of the new substitutions on the key codons is statistically related to the subtype positivity rate, these important amino acid sites are denoted as EM sites, which can be used to measure genetic distance.

**S2 Supplementary figure**

## Figure S2.1. The relationship between H1N1 VE and EMD on neuraminidase (NA), in different age groups.


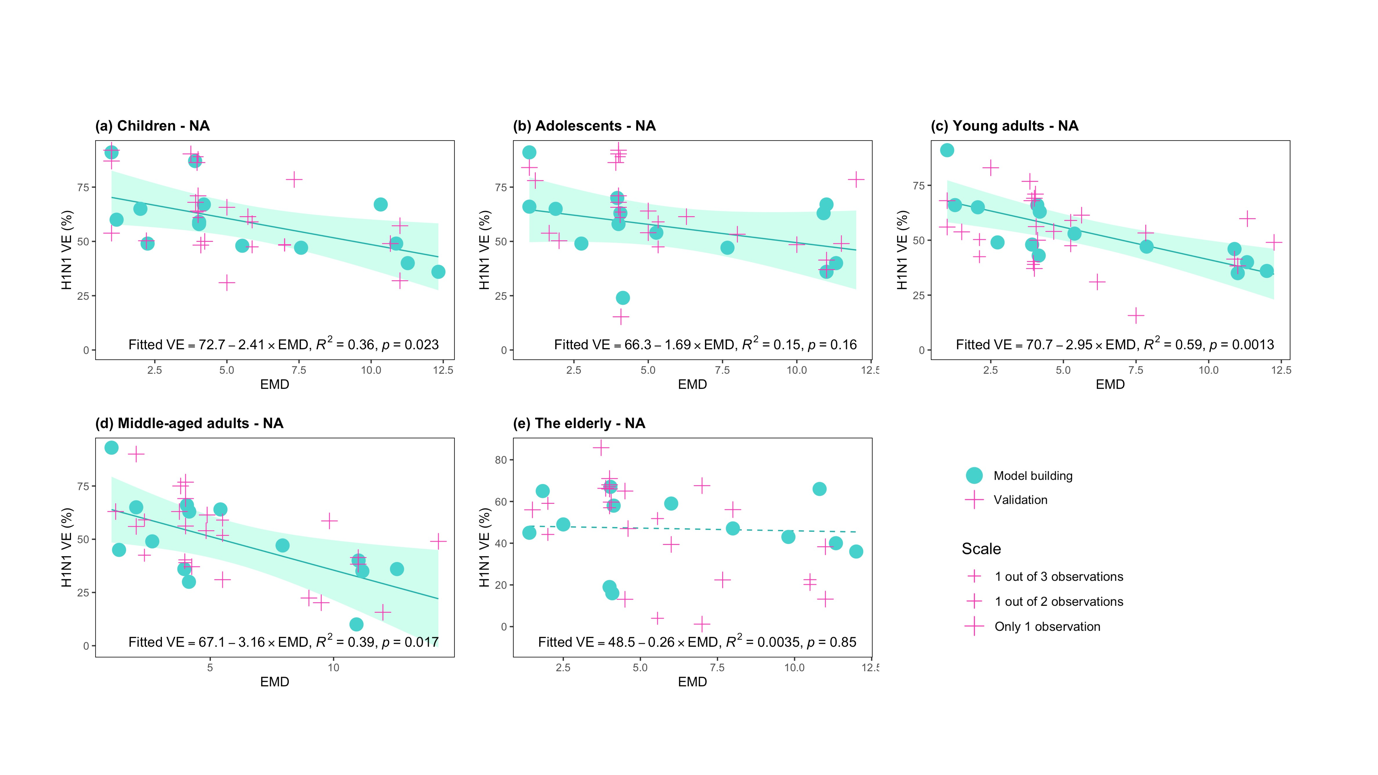


Legend: Similar to HA results, for the first four age groups, clear linear relationship were observed between VE and the EMD; the validation samples largely followed the relationship identified for North American samples (a, b, c and d). For the elderly population, no evident association was identified between VE and the EMD (e).

**References**

1. Treanor, J. J.; Talbot, H. K.; Ohmit, S. E.; Coleman, L. A.; Thompson, M. G.; Cheng, P. Y.; Petrie, J. G.; Lofthus, G.; Meece, J. K.; Williams, J. V.; Berman, L.; Breese Hall, C.; Monto, A. S.; Griffin, M. R.; Belongia, E.; Shay, D. K.; Network, U. S. F.-V., Effectiveness of seasonal influenza vaccines in the United States during a season with circulation of all three vaccine strains. *Clin Infect Dis* **2012,** 55, (7), 951-9.

2. Ohmit, S. E.; Thompson, M. G.; Petrie, J. G.; Thaker, S. N.; Jackson, M. L.; Belongia, E. A.; Zimmerman, R. K.; Gaglani, M.; Lamerato, L.; Spencer, S. M.; Jackson, L.; Meece, J. K.; Nowalk, M. P.; Song, J.; Zervos, M.; Cheng, P. Y.; Rinaldo, C. R.; Clipper, L.; Shay, D. K.; Piedra, P.; Monto, A. S., Influenza vaccine effectiveness in the 2011-2012 season: protection against each circulating virus and the effect of prior vaccination on estimates. *Clin Infect Dis* **2014,** 58, (3), 319-27.

3. McLean, H. Q.; Thompson, M. G.; Sundaram, M. E.; Kieke, B. A.; Gaglani, M.; Murthy, K.; Piedra, P. A.; Zimmerman, R. K.; Nowalk, M. P.; Raviotta, J. M.; Jackson, M. L.; Jackson, L.; Ohmit, S. E.; Petrie, J. G.; Monto, A. S.; Meece, J. K.; Thaker, S. N.; Clippard, J. R.; Spencer, S. M.; Fry, A. M.; Belongia, E. A., Influenza vaccine effectiveness in the United States during 2012-2013: variable protection by age and virus type. *J Infect Dis* **2015,** 211, (10), 1529-40.

4. Gaglani, M.; Pruszynski, J.; Murthy, K.; Clipper, L.; Robertson, A.; Reis, M.; Chung, J. R.; Piedra, P. A.; Avadhanula, V.; Nowalk, M. P.; Zimmerman, R. K.; Jackson, M. L.; Jackson, L. A.; Petrie, J. G.; Ohmit, S. E.; Monto, A. S.; McLean, H. Q.; Belongia, E. A.; Fry, A. M.; Flannery, B., Influenza Vaccine Effectiveness Against 2009 Pandemic Influenza A(H1N1) Virus Differed by Vaccine Type During 2013-2014 in the United States. *J Infect Dis* **2016,** 213, (10), 1546-56.

5. Zimmerman, R. K.; Nowalk, M. P.; Chung, J.; Jackson, M. L.; Jackson, L. A.; Petrie, J. G.; Monto, A. S.; McLean, H. Q.; Belongia, E. A.; Gaglani, M.; Murthy, K.; Fry, A. M.; Flannery, B.; Investigators, U. S. F. V.; Investigators, U. S. F. V., 2014-2015 Influenza Vaccine Effectiveness in the United States by Vaccine Type. *Clin Infect Dis* **2016,** 63, (12), 1564-1573.

6. Jackson, M. L.; Chung, J. R.; Jackson, L. A.; Phillips, C. H.; Benoit, J.; Monto, A. S.; Martin, E. T.; Belongia, E. A.; McLean, H. Q.; Gaglani, M.; Murthy, K.; Zimmerman, R.; Nowalk, M. P.; Fry, A. M.; Flannery, B., Influenza Vaccine Effectiveness in the United States during the 2015-2016 Season. *N Engl J Med* **2017,** 377, (6), 534-543.

7. Flannery, B.; Chung, J. R.; Monto, A. S.; Martin, E. T.; Belongia, E. A.; McLean, H. Q.; Gaglani, M.; Murthy, K.; Zimmerman, R. K.; Nowalk, M. P.; Jackson, M. L.; Jackson, L. A.; Rolfes, M. A.; Spencer, S.; Fry, A. M.; Investigators, U. S. F. V., Influenza Vaccine Effectiveness in the United States During the 2016-2017 Season. *Clin Infect Dis* **2019,** 68, (11), 1798-1806.

8. Rolfes, M. A.; Flannery, B.; Chung, J. R.; O'Halloran, A.; Garg, S.; Belongia, E. A.; Gaglani, M.; Zimmerman, R. K.; Jackson, M. L.; Monto, A. S.; Alden, N. B.; Anderson, E.; Bennett, N. M.; Billing, L.; Eckel, S.; Kirley, P. D.; Lynfield, R.; Monroe, M. L.; Spencer, M.; Spina, N.; Talbot, H. K.; Thomas, A.; Torres, S. M.; Yousey-Hindes, K.; Singleton, J. A.; Patel, M.; Reed, C.; Fry, A. M., Effects of Influenza Vaccination in the United States During the 2017-2018 Influenza Season. *Clin Infect Dis* **2019,** 69, (11), 1845-1853.

9. Flannery, B.; Kondor, R. J. G.; Chung, J. R.; Gaglani, M.; Reis, M.; Zimmerman, R. K.; Nowalk, M. P.; Jackson, M. L.; Jackson, L. A.; Monto, A. S.; Martin, E. T.; Belongia, E. A.; McLean, H. Q.; Kim, S. S.; Blanton, L.; Kniss, K.; Budd, A. P.; Brammer, L.; Stark, T. J.; Barnes, J. R.; Wentworth, D. E.; Fry, A. M.; Patel, M., Spread of Antigenically Drifted Influenza A(H3N2) Viruses and Vaccine Effectiveness in the United States During the 2018-2019 Season. *J Infect Dis* **2020,** 221, (1), 8-15.

10. Skowronski, D. M.; Janjua, N. Z.; De Serres, G.; Hottes, T. S.; Dickinson, J. A.; Crowcroft, N.; Kwindt, T. L.; Tang, P.; Charest, H.; Fonseca, K.; Gubbay, J. B.; Bastien, N.; Li, Y.; Petric, M., Effectiveness of AS03 adjuvanted pandemic H1N1 vaccine: case-control evaluation based on sentinel surveillance system in Canada, autumn 2009. *Bmj* **2011,** 342, c7297.

11. Skowronski, D. M.; Chambers, C.; Sabaiduc, S.; De Serres, G.; Winter, A. L.; Dickinson, J. A.; Gubbay, J. B.; Drews, S. J.; Martineau, C.; Charest, H.; Krajden, M.; Bastien, N.; Li, Y., Beyond Antigenic Match: Possible Agent-Host and Immuno-epidemiological Influences on Influenza Vaccine Effectiveness During the 2015-2016 Season in Canada. *J Infect Dis* **2017,** 216, (12), 1487-1500.

12. Skowronski, D. M.; Leir, S.; Sabaiduc, S.; Chambers, C.; Zou, M.; Rose, C.; Olsha, R.; Dickinson, J. A.; Winter, A. L.; Jassem, A.; Gubbay, J. B.; Drews, S. J.; Charest, H.; Chan, T.; Hickman, R.; Bastien, N.; Li, Y.; Krajden, M.; De Serres, G., Influenza vaccine effectiveness by A(H3N2) phylogenetic sub-cluster and prior vaccination history: 2016-17 and 2017-18 epidemics in Canada. *J Infect Dis* **2020**.

13. Skowronski, D. M.; Sabaiduc, S.; Leir, S.; Rose, C.; Zou, M.; Murti, M.; Dickinson, J. A.; Olsha, R.; Gubbay, J. B.; Croxen, M. A.; Charest, H.; Bastien, N.; Li, Y.; Jassem, A.; Krajden, M.; De Serres, G., Paradoxical clade- and age-specific vaccine effectiveness during the 2018/19 influenza A(H3N2) epidemic in Canada: potential imprint-regulated effect of vaccine (I-REV). *Euro Surveill* **2019,** 24, (46).

14. Pebody, R. G.; Andrews, N.; Fleming, D. M.; McMenamin, J.; Cottrell, S.; Smyth, B.; Durnall, H.; Robertson, C.; Carman, W.; Ellis, J.; Sebastian-Pillai, P.; Zambon, M.; Kearns, C.; Moore, C.; Thomas, D. R.; Watson, J. M., Age-specific vaccine effectiveness of seasonal 2010/2011 and pandemic influenza A(H1N1) 2009 vaccines in preventing influenza in the United Kingdom. *Epidemiol Infect* **2013,** 141, (3), 620-30.

15. Andrews, N.; McMenamin, J.; Durnall, H.; Ellis, J.; Lackenby, A.; Robertson, C.; von Wissmann, B.; Cottrell, S.; Smyth, B.; Moore, C.; Gunson, R.; Zambon, M.; Fleming, D.; Pebody, R., Effectiveness of trivalent seasonal influenza vaccine in preventing laboratory-confirmed influenza in primary care in the United Kingdom: 2012/13 end of season results. *Euro Surveill* **2014,** 19, (27), 5-13.

16. Pebody, R.; Warburton, F.; Ellis, J.; Andrews, N.; Potts, A.; Cottrell, S.; Johnston, J.; Reynolds, A.; Gunson, R.; Thompson, C.; Galiano, M.; Robertson, C.; Byford, R.; Gallagher, N.; Sinnathamby, M.; Yonova, I.; Pathirannehelage, S.; Donati, M.; Moore, C.; de Lusignan, S.; McMenamin, J.; Zambon, M., Effectiveness of seasonal influenza vaccine for adults and children in preventing laboratory-confirmed influenza in primary care in the United Kingdom: 2015/16 end-of-season results. *Euro Surveill* **2016,** 21, (38).

17. Pebody, R.; Sile, B.; Warburton, F.; Sinnathamby, M.; Tsang, C.; Zhao, H.; Ellis, J.; Andrews, N., Live attenuated influenza vaccine effectiveness against hospitalisation due to laboratory-confirmed influenza in children two to six years of age in England in the 2015/16 season. *Euro Surveill* **2017,** 22, (4).

18. England, P. H., Influenza vaccine effectiveness (VE) in adults and children in primary care in the United Kingdom (UK): provisional end-of-season results 2017-18. In The Stationery Office London: 2018.

19. Kissling, E.; Rose, A.; Emborg, H. D.; Gherasim, A.; Pebody, R.; Pozo, F.; Trebbien, R.; Mazagatos, C.; Whitaker, H.; Valenciano, M., Interim 2018/19 influenza vaccine effectiveness: six European studies, October 2018 to January 2019. *Euro Surveill* **2019,** 24, (8).

20. Pebody, R. G.; Zhao, H.; Whitaker, H. J.; Ellis, J.; Donati, M.; Zambon, M.; Andrews, N., Effectiveness of influenza vaccine in children in preventing influenza associated hospitalisation, 2018/19, England. *Vaccine* **2020,** 38, (2), 158-164.

21. Pebody, R. G.; Whitaker, H.; Ellis, J.; Andrews, N.; Marques, D. F. P.; Cottrell, S.; Reynolds, A. J.; Gunson, R.; Thompson, C.; Galiano, M.; Lackenby, A.; Robertson, C.; O'Doherty, M. G.; Owens, K.; Yonova, I.; Shepherd, S. J.; Moore, C.; Johnston, J.; Donati, M.; McMenamin, J.; Lusignan, S.; Zambon, M., End of season influenza vaccine effectiveness in primary care in adults and children in the United Kingdom in 2018/19. *Vaccine* **2020,** 38, (3), 489-497.

22. Kissling, E.; Valenciano, M.; Cohen, J. M.; Oroszi, B.; Barret, A. S.; Rizzo, C.; Stefanoff, P.; Nunes, B.; Pitigoi, D.; Larrauri, A.; Daviaud, I.; Horvath, J. K.; O'Donnell, J.; Seyler, T.; Paradowska-Stankiewicz, I. A.; Pechirra, P.; Ivanciuc, A. E.; Jimenez-Jorge, S.; Savulescu, C.; Ciancio, B. C.; Moren, A., I-MOVE multi-centre case control study 2010-11: overall and stratified estimates of influenza vaccine effectiveness in Europe. *PLoS One* **2011,** 6, (11), e27622.

23. Kissling, E.; Valenciano, M.; Buchholz, U.; Larrauri, A.; Cohen, J. M.; Nunes, B.; Rogalska, J.; Pitigoi, D.; Paradowska-Stankiewicz, I.; Reuss, A.; Jimenez-Jorge, S.; Daviaud, I.; Guiomar, R.; O'Donnell, J.; Necula, G.; Gluchowska, M.; Moren, A., Influenza vaccine effectiveness estimates in Europe in a season with three influenza type/subtypes circulating: the I-MOVE multicentre case-control study, influenza season 2012/13. *Euro Surveill* **2014,** 19, (6).

24. Valenciano, M.; Kissling, E.; Reuss, A.; Jimenez-Jorge, S.; Horvath, J. K.; Donnell, J. M.; Pitigoi, D.; Machado, A.; Pozo, F., The European I-MOVE Multicentre 2013-2014 Case-Control Study. Homogeneous moderate influenza vaccine effectiveness against A(H1N1)pdm09 and heterogenous results by country against A(H3N2). *Vaccine* **2015,** 33, (24), 2813-22.

25. Kissling, E.; Valenciano, M.; Pozo, F.; Vilcu, A. M.; Reuss, A.; Rizzo, C.; Larrauri, A.; Horvath, J. K.; Brytting, M.; Domegan, L.; Korczynska, M.; Meijer, A.; Machado, A.; Ivanciuc, A.; Visekruna Vucina, V.; van der Werf, S.; Schweiger, B.; Bella, A.; Gherasim, A.; Ferenczi, A.; Zakikhany, K.; J, O. D.; Paradowska-Stankiewicz, I.; Dijkstra, F.; Guiomar, R.; Lazar, M.; Kurecic Filipovic, S.; Johansen, K.; Moren, A., 2015/16 I-MOVE/I-MOVE+ multicentre case-control study in Europe: Moderate vaccine effectiveness estimates against influenza A(H1N1)pdm09 and low estimates against lineage-mismatched influenza B among children. *Influenza Other Respir Viruses* **2018,** 12, (4), 423-437.

26. Rondy, M.; Kissling, E.; Emborg, H. D.; Gherasim, A.; Pebody, R.; Trebbien, R.; Pozo, F.; Larrauri, A.; McMenamin, J.; Valenciano, M., Interim 2017/18 influenza seasonal vaccine effectiveness: combined results from five European studies. *Euro Surveill* **2018,** 23, (9).

27. Kissling, E.; Nunes, B.; Robertson, C.; Valenciano, M.; Reuss, A.; Larrauri, A.; Cohen, J. M.; Oroszi, B.; Rizzo, C.; Machado, A.; Pitigoi, D.; Domegan, L.; Paradowska-Stankiewicz, I.; Buchholz, U.; Gherasim, A.; Daviaud, I.; Horváth, J. K.; Bella, A.; Lupulescu, E.; J, O. D.; Korczyńska, M.; Moren, A., I-MOVE multicentre case-control study 2010/11 to 2014/15: Is there within-season waning of influenza type/subtype vaccine effectiveness with increasing time since vaccination? *Euro Surveill* **2016,** 21, (16).

28. Rondy, M.; Launay, O.; Puig-Barberà, J.; Gefenaite, G.; Castilla, J.; de Gaetano Donati, K.; Galtier, F.; Hak, E.; Guevara, M.; Costanzo, S.; Moren, A., 2012/13 influenza vaccine effectiveness against hospitalised influenza A(H1N1)pdm09, A(H3N2) and B: estimates from a European network of hospitals. *Euro Surveill* **2015,** 20, (2).

29. Castilla, J.; Martínez-Baz, I.; Navascués, A.; Fernandez-Alonso, M.; Reina, G.; Guevara, M.; Chamorro, J.; Ortega, M. T.; Albéniz, E.; Pozo, F.; Ezpeleta, C., Vaccine effectiveness in preventing laboratory-confirmed influenza in Navarre, Spain: 2013/14 mid-season analysis. *Euro Surveill* **2014,** 19, (6).

30. Puig-Barberà, J.; Guglieri-López, B.; Tortajada-Girbés, M.; López-Labrador, F. X.; Carballido-Fernández, M.; Mollar-Maseres, J.; Schwarz-Chavarri, G.; Baselga-Moreno, V.; Mira-Iglesias, A.; Díez-Domingo, J., Low influenza vaccine effectiveness and the effect of previous vaccination in preventing admission with A(H1N1)pdm09 or B/Victoria-Lineage in patients 60 years old or older during the 2015/2016 influenza season. *Vaccine* **2017,** 35, (52), 7331-7338.

31. Rondy, M.; Larrauri, A.; Casado, I.; Alfonsi, V.; Pitigoi, D.; Launay, O.; Syrjänen, R. K.; Gefenaite, G.; Machado, A.; Vučina, V. V.; Horváth, J. K.; Paradowska-Stankiewicz, I.; Marbus, S. D.; Gherasim, A.; Díaz-González, J. A.; Rizzo, C.; Ivanciuc, A. E.; Galtier, F.; Ikonen, N.; Mickiene, A.; Gomez, V.; Kurečić Filipović, S.; Ferenczi, A.; Korcinska, M. R.; van Gageldonk-Lafeber, R.; Valenciano, M., 2015/16 seasonal vaccine effectiveness against hospitalisation with influenza A(H1N1)pdm09 and B among elderly people in Europe: results from the I-MOVE+ project. *Euro Surveill* **2017,** 22, (30).

32. Kissling, E.; Pozo, F.; Buda, S.; Vilcu, A. M.; Rizzo, C.; Gherasim, A.; Horváth, J. K.; Brytting, M.; Domegan, L.; Meijer, A.; Paradowska-Stankiewicz, I.; Machado, A.; Vučina, V. V.; Lazar, M.; Johansen, K.; Dürrwald, R.; van der Werf, S.; Bella, A.; Larrauri, A.; Ferenczi, A.; Zakikhany, K.; O'Donnell, J.; Dijkstra, F.; Bogusz, J.; Guiomar, R.; Filipović, S. K.; Pitigoi, D.; Penttinen, P.; Valenciano, M., Effectiveness of influenza vaccine against influenza A in Europe in seasons of different A(H1N1)pdm09 and the same A(H3N2) vaccine components (2016-17 and 2017-18). *Vaccine X* **2019,** 3, 100042.

33. Castilla, J.; Portillo, M. E.; Casado, I.; Pozo, F.; Navascués, A.; Adelantado, M.; Gómez Ibáñez, C.; Ezpeleta, C.; Martínez-Baz, I., Effectiveness of the current and prior influenza vaccinations in Northern Spain, 2018-2019. *Vaccine* **2020,** 38, (8), 1925-1932.

34. Rizzo, C.; Bella, A.; Alfonsi, V.; Puzelli, S.; Palmieri, A. P.; Chironna, M.; Pariani, E.; Piatti, A.; Tiberti, D.; Ghisetti, V.; Rangoni, R.; Colucci, M. E.; Affanni, P.; Germinario, C.; Castrucci, M. R., Influenza vaccine effectiveness in Italy: Age, subtype-specific and vaccine type estimates 2014/15 season. *Vaccine* **2016,** 34, (27), 3102-3108.

35. Bella, A.; Gesualdo, F.; Orsi, A.; Arcuri, C.; Chironna, M.; Loconsole, D.; Napoli, C.; Orsi, G. B.; Manini, I.; Montomoli, E.; Alfonsi, V.; Castrucci, M. R.; Rizzo, C., Effectiveness of the trivalent MF59 adjuvated influenza vaccine in preventing hospitalization due to influenza B and A(H1N1)pdm09 viruses in the elderly in Italy, 2017 - 2018 season. *Expert Rev Vaccines* **2019,** 18, (6), 671-679.

36. Bellino, S.; Bella, A.; Puzelli, S.; Di Martino, A.; Facchini, M.; Punzo, O.; Pezzotti, P.; Castrucci, M. R.; The InfluNet Study, G., Moderate influenza vaccine effectiveness against A(H1N1)pdm09 virus, and low effectiveness against A(H3N2) subtype, 2018/19 season in Italy. *Expert Rev Vaccines* **2019,** 18, (11), 1201-1209.

37. Rondy, M.; Castilla, J.; Launay, O.; Costanzo, S.; Ezpeleta, C.; Galtier, F.; de Gaetano Donati, K.; Moren, A., Moderate influenza vaccine effectiveness against hospitalisation with A(H3N2) and A(H1N1) influenza in 2013-14: Results from the InNHOVE network. *Hum Vaccin Immunother* **2016,** 12, (5), 1217-24.

38. Souty, C.; Vilcu, A. M.; Capai, L.; van der Werf, S.; Valette, M.; Blanchon, T.; Lina, B.; Behillil, S.; Hanslik, T.; Falchi, A., Early estimates of 2016/17 seasonal influenza vaccine effectiveness in primary care in France. *J Clin Virol* **2017,** 95, 1-4.

39. Lytras, T.; Kossyvakis, A.; Melidou, A.; Exindari, M.; Gioula, G.; Pogka, V.; Malisiovas, N.; Mentis, A., Influenza vaccine effectiveness against laboratory confirmed influenza in Greece during the 2013-2014 season: a test-negative study. *Vaccine* **2015,** 33, (2), 367-73.

40. Widgren, K.; Magnusson, M.; Hagstam, P.; Widerström, M.; Örtqvist, Å.; Einemo, I. M.; Follin, P.; Lindblom, A.; Mäkitalo, S.; Wik, O.; Österlund, A.; Grünewald, M.; Uhnoo, I.; Linde, A., Prevailing effectiveness of the 2009 influenza A(H1N1)pdm09 vaccine during the 2010/11 season in Sweden. *Euro Surveill* **2013,** 18, (15), 20447.

41. Castillejos, M.; Cabello-Gutiérrez, C.; Alberto Choreño-Parra, J.; Hernández, V.; Romo, J.; Hernández-Sánchez, F.; Martínez, D.; Hernández, A.; Jiménez-Álvarez, L.; Hernández-Cardenas, C. M.; Becerril-Vargas, E.; Martínez-Orozco, J. A.; Luis Sandoval-Gutiérrez, J.; Guadarrama, C.; Olvera-Masetto, E.; Alfaro-Ramos, L.; Cruz-Lagunas, A.; Ramírez, G.; Márquez, E.; Pimentel, L.; Regino-Zamarripa, N. E.; Mendoza-Milla, C.; Goodina, A.; Hernández-Montiel, E.; Barquera, R.; Santibañez, A.; Domínguez-Cherit, G.; Pérez-Padilla, R.; Regalado, J.; Santillán-Doherty, P.; Salas-Hernández, J.; Zúñiga, J., High performance of rapid influenza diagnostic test and variable effectiveness of influenza vaccines in Mexico. *Int J Infect Dis* **2019,** 89, 87-95.

42. Ando, S., Estimation of the Effectiveness of Quadrivalent Influenza Vaccines by Distinguishing Between Influenza A (H1N1) pdm09 and Influenza A (H3N2) Using Rapid Influenza Diagnostic Tests During the 2018-2019 Season. *Intern Med* **2020,** 59, (7), 933-940.

43. Chan, Y. D.; Wong, M. L.; Au, K. W.; Chuang, S. K., Seasonal influenza vaccine effectiveness at primary care level, Hong Kong SAR, 2017/2018 winter. *Hum Vaccin Immunother* **2019,** 15, (1), 97-101.

44. Chiu, S. S.; Kwan, M. Y.; Feng, S.; Chan, E. L.; Chua, H.; Wong, J. S.; Peiris, J. M.; Cowling, B. J., Early season estimate of influenza vaccination effectiveness against influenza hospitalisation in children, Hong Kong, winter influenza season 2018/19. *Euro Surveill* **2019,** 24, (5).

45. Wang, M. H.; Lou, J.; Cao, L.; Zhao, S.; Chan, P. K.; Chan, M. C.; Chong, M. K.; Wu, W. K. K.; Chan, R. W.; Wei, Y.; Zhang, H.; Zee, B. C.; Yeoh, E.-k., Characterization of the evolutionary dynamics of influenza A H3N2 hemagglutinin. *BioRxiv* **2020**.

46. Zhao, S.; Lou, J.; Cao, L.; Chen, Z.; Chan, R. W.; Chong, M. K.; Zee, B. C.; Chan, P. K.; Wang, M. H., Quantifying the importance of the key sites on haemagglutinin in determining the selection advantage of influenza virus: using A/H3N2 as an example. *J Infect* **2020**.

47. Wang, M. H.; Zee, B. C.; Lou, J.; Chong, M. K. Measurement and Prediction on Influenza Virus Genetic Mutation Patterns. PCT/CN2019/091652, 2019.

48. Lou, J.; Zhao, S.; Cao, L.; Chong, M. K. C.; Chan, R. W. Y.; Chan, P. K. S.; Zee, B. C. Y.; Yeoh, E. K.; Wang, M. H., Predicting the dominant influenza A serotype by quantifying mutation activities. *Int J Infect Dis* **2020,** 100, 255-257.
